# Fermentation Technologies to Produce and Improve Alternative Protein Sources

**DOI:** 10.3390/foods15010117

**Published:** 2025-12-31

**Authors:** Jonathan Coronel-León, Daniela Maza, Ignacio García-Álvarez de Toledo, Anna Jofré, Belén Martín, Xavier Serra, Sara Bover-Cid

**Affiliations:** 1 Food Safety and Functionality Program, Institute of Agrifood Research and Technology (IRTA), Finca Camps i Armet, 17121 Monells, Spain; daniela.maza@irta.cat (D.M.); ignacio.garcia@irta.cat (I.G.-Á.d.T.); anna.jofre@irta.cat (A.J.); belen.martin@irta.cat (B.M.); 2 Facultad de Ingeniería Mecánica y Ciencias de la Producción (FIMCP), Centro de Investigaciones Biotecnológicas del Ecuador (CIBE), Escuela Superior Politécnica del Litoral, ESPOL, Campus Gustavo Galindo Km, 30.5 Vía Perimetral, Guayaquil 090902, Ecuador; 3 Food Quality and Technology Program, Institute of Agrifood Research and Technology (IRTA), Finca Camps i Armet, 17121 Monells, Spain; xavier.serra@irta.cat

**Keywords:** high-value ingredient, microbial strategies, microbial food cultures, biomass-derived ingredients, microbial metabolic pathways, food innovation

## Abstract

The growing global population, along with evolving dietary trends and increasing concerns about health and the environment, underscores the urgent need to transform current food systems to minimize their environmental footprint and enhance global food security. This transformation has driven the development and demand for alternative food sources. In this context, alternative proteins emerge as promising options due to their production from plants, microorganisms, and insects, which potentially reduces the environmental impact of food production while supporting global food security. Nevertheless, the transition toward alternative proteins presents significant challenges related to the presence of antinutritional compounds, poor amino acid composition, lower digestibility, and undesirable organoleptic characteristics. Moreover, these new generations of alternative foods are highly processed, raising concerns about their nutritional adequacy compared to traditional products. In this context, fermentation technologies have emerged as promising tools to overcome these limitations. Traditional fermentation can degrade antinutritional factors, improve digestibility, and release bioactive compounds, allowing the production of new products with health-promoting properties. Beyond traditional fermentation, biomass fermentation to single-cell protein or microbial protein production represents a sustainable alternative, promoting a climate-friendly approach aligned with circular bioeconomy principles by upcycling various agro-industrial streams. Thus, this review discusses how microbial strategies (from traditional fermentation to cutting-edge microbial protein production) can enhance the nutritional properties of alternative protein-based foods. Emphasis is placed on the capacity of traditional fermentation to improve nutritional quality and bioactivity, mitigate undesirable sensory traits, and preserve or enhance micronutrient content. Additionally, integrating biomass fermentation and emerging precision fermentation positions microorganisms as valuable contributors to more nutritious and sustainable food systems.

## 1. Introduction

The global population is growing exponentially; according to projections by the United Nations, the world population will reach 9.7 billion by 2050 [1]. This demographic expansion has profound implications for food security, with an estimated 295.3 million people already facing high levels of acute food insecurity, underscoring the persistent challenge of ensuring access to nutritionally adequate diets [2]. Thus, feeding future generations in a manner that is both nutritious and sustainable, and equitable, constitutes one of the most pressing challenges facing global food systems. Food products provide essential macronutrients, minerals, and phytochemicals, which can be consumed either in their fresh or processed form. Among these, proteins occupy a central role as an essential macronutrient, as they supply indispensable amino acids (IAA) that are critical for human health. Proteins are structural components (supporting growth by tissue building and repair, and the maintenance of body cell mass), enzymes and hormones catalyzing biochemical and physiological reactions, contribute to immune function (e.g., antibodies), transport and storage functions (e.g., hemoglobin as an oxygen carrier), and are an energy source when needed [3,4]. Historically, animal-derived proteins have been considered a primary dietary source due to their high-quality protein content and complete essential amino acid profiles, which meet human requirements. Furthermore, animal-derived proteins are known to be digested and absorbed more efficiently than those from many plant sources. For instance, Marinangeli et al. [5] reported that approximately 95–100% of animal proteins are digested in the human gastrointestinal tract, compared with about 80–95% for plant proteins, depending on their origin. However, significant disparities exist in the production and consumption of protein sources across countries because of differences in economic development, food availability, agricultural practices, and cultural dietary habits [6]. As reviewed by Salter et al. [7] in Africa, cereals such as wheat, rice, and maize dominate protein intake, reflecting both agricultural traditions and economic constraints that limit access to animal-based protein. Similarly, in Asia, cereals remain the principal source of protein, complemented by moderate contributions from fish and seafood, largely driven by the region’s strong coastal and aquaculture sectors. In contrast, the Americas and Europe exhibit greater reliance on animal-derived proteins, particularly meat, dairy, and eggs, a trend linked to higher economic prosperity, cultural preferences, and the intensive livestock sector. Oceania stands out for its particularly high meat consumption, which is consistent with its robust livestock production systems and dietary culture. In recent decades, emerging and increasing dietary trends such as flexitarianism (focusing on reducing rather than eliminating meat consumption), vegetarianism, and veganism demonstrate that significant differences in protein source preferences among the population are also evident within countries, further diversifying global dietary patterns [4,8].

It is essential to note that the fulfillment of dietary protein requirements is influenced by both the quality and quantity of protein in food. The World Health Organization (WHO) recommends prioritizing protein sources with a complete amino acid profile and high digestibility, typically of animal origin, as they provide the full spectrum of indispensable amino acids and play a crucial role in meeting human protein needs [9,10]. These facts highlight that the goal is not necessarily to eliminate animal-derived foods, but to decrease excessive consumption of animal-based foods associated with negative health impacts, as well as to seek sustainable alternatives that reduce environmental impact in producing enough food for the growing population, while addressing the diverse needs and preferences of consumers [11]. As a result, the global food industry has undergone a significant transformation, exploring alternative protein sources, driven by increasing demand for protein transition and diversification [12]. Additionally, increasing attention is being directed not only to the quantity and quality of protein consumed but also to how food is produced, processed, and its broader environmental implications. The ecological burden of conventional animal-based food production systems, including greenhouse gas emissions, land and water use, and biodiversity loss, has raised concerns among scientists, policymakers, and consumers alike [13].

Alternative proteins are defined as protein sources that do not originate from conventional livestock products but are derived from plants, microorganisms, algae, and insects, offering potentially more sustainable options for human and animal diets [14]. In the European context, these proteins are classified into three main categories: (1) plant-based alternatives ranging from minimally processed legumes to highly engineered products designed to mimic meat and dairy; (2) non-plant alternatives that are relatively new to the EU but have a long history of use in other cultures, including algae and insects; and (3) entirely novel alternatives, such as microbial fermentation and cultured meat, which have only recently emerged as potential food sources [15]. Political and institutional efforts are already underway to support protein transition. For instance, the European Union’s Food 2030 research and innovation policy framework supports the shift towards sustainable, healthy, and inclusive food systems, identifies alternative proteins as one of the key action pathways to achieve food system resilience and sustainability [16]. While alternative proteins represented 2% of the global consumption of protein products in 2020, overall industry trends and market predictions indicate substantial growth in the global alternative protein market worldwide, projected to reach USD 290 billion by 2035, accounting for approximately 11% of the total protein market under a base case scenario [17,18].

This expansion is driven by rising consumer interest in plant-based diets, rapid technological innovations in food production, and a surge in investment, with billions of dollars poured into the sector and hundreds of new companies entering the market [18]. In this context, legumes, cereals, pseudocereals, and seeds are attractive protein sources due to their high nutrient density, fiber content, and phytochemical compounds [12]. However, the variability in nutritional quality among plant-based protein sources must be carefully considered when developing new products, particularly to ensure adequate dietary protein intake in more vulnerable populations.

The quality of plant-based foods can be much more variable, both because of lower IAA contents and poorer digestibility, often associated with the presence of bioactive compounds (antinutritional factors, ANFs) such as trypsin inhibitors, phytic acid, tannins, and raffinose families that impair nutrient bioavailability, protein digestibility, and generate unfavorable sensory attributes [7,9,19]. In addition, many next-generation plant-based products are usually also highly processed, raising concerns about their nutritional adequacy and long-term health implications. Therefore, traditional and modern processing techniques have been applied to tackle these issues. Methods like soaking, cooking, roasting, germination, and dehulling help reduce heat-sensitive ANFs. More advanced techniques, such as extrusion, high-pressure processing, ultrasound, and enzymatic hydrolysis, address some techno-functional limitations but often fail to eliminate thermostable compounds, including phytic acid and tannins [20,21,22]. Alternatively, biological interventions, particularly fermentation, have demonstrated superior efficacy in improving the nutritional and sensory qualities of alternative proteins [23,24].

The fermentation process is defined as the chemical transformation of organic matter via microbial metabolism mediated by enzymes, which, in a plant-based context, can significantly enhance digestibility, reduce or eliminate ANFs, and enrich the protein matrix with bioactive molecules such as peptides, phenolics, and short-chain fatty acids [25]. In this line, microbial strategies such as traditional fermentation (TF), biomass fermentation (BF), and precision fermentation (PF) are now harnessed in the alternative protein industry to produce innovative ingredients and foods [26]. Indeed, TF supports the production of products such as tofu, tempeh, and natto, which boast unique flavors, textures, and nutritional profiles. BF harnesses the rapid growth and high protein content of microorganisms, including filamentous fungi, yeast, microalgae, and bacteria, to produce substantial quantities of microbial protein (MP) or single-cell protein (SCP). In contrast, PF utilizes genetically engineered microorganisms to produce targeted, high-value compounds, including proteins, enzymes, vitamins, pigments, and fats, thereby enhancing the sensory and functional attributes of plant-based foods [23,27]. Furthermore, the versatility of some microorganisms allows them to grow on complex substrates. In this context, SCP production could be supported by approximately 1.3 billion tons of food that are lost or wasted annually, containing nutrients such as carbohydrates, proteins, lipids, and other micronutrients [28], thus contributing to the valorization of agro-industrial wastes or by-products and fostering the development of sustainable circular bioeconomy frameworks.

The objective of this review is to provide an updated and integrated overview of how microbial fermentation technologies, from TF and BF to PF, can be applied to enhance and diversify alternative protein ingredients. In this context, this review first describes the characteristics of the plant-based proteins most used as ingredients in the formulation of new plant-based food products. Then, it provides a comprehensive overview of how TF can address its inherent limitations in terms of nutritional quality. Furthermore, this review examines the application of BF in producing single-cell proteins, a promising alternative. Finally, future trends related to the use of PF to modify microorganisms for enhanced production of specific compounds, thereby supporting the development of healthier, more widely accepted, and environmentally sustainable food products, are discussed. The literature included in this review was identified through searches conducted in Google Scholar, PubMed, Scopus, and ScienceDirect using keyword combinations related to plant-based proteins and fermentation technologies.

## 2. Plant-Based Proteins: Importance, Advantages, and Limitations

The development of alternative proteins, particularly those derived from plants, has gained significant interest in recent years. This surge is driven by various factors, from shifting consumer perspectives to pressing environmental concerns. For instance, health-related events such as the COVID-19 pandemic have further heightened consumer interest in exploring food alternatives that meet nutritional requirements and support the prevention or management of health conditions. These evolving demands have been acknowledged not only by the food industry but also by governments worldwide. As a result of this growing interest, numerous studies have explored the use of diverse plant-based raw materials for producing food ingredients and final products, including protein isolates or concentrates, as well as analog products mimicking meat, fish, eggs, and dairy products. Food processors and researchers have increasingly focused on plant matrices with high protein content. As a result, the commercialization of plant protein ingredients has shown steady growth, rising from USD 6.7 billion in 2020 to a projected USD 23.0 billion by 2035. With a compound annual growth rate (CAGR) of 8.6% between 2025 and 2035 [29]. This trend highlights the increasing global demand for healthier and more sustainable alternatives, representing a nutritional and sustainable raw material for the food industry [30].

However, it is crucial to recognize that plant proteins differ significantly from animal proteins in terms of nutritional profile, food safety hazards, technological characteristics, and sensory characteristics. These differences highlight the need for a comprehensive evaluation of plant protein quality to ensure that its nutritional and functional potential is properly understood and optimized. In this context, ANFs are synthesized as secondary metabolites by plants and are responsible for several undesirable effects in plant-based ingredients, which limit their application in developing alternative protein-based products. ANFs include protease and amylase inhibitors, phytic acid, saponins, alkaloids, lectins, certain oligosaccharides, glucosinolates, tannins, phytoestrogens, and cyanogenic glycosides, which are considered non-beneficial compounds. They can alter the digestion, absorption, and utilization of proteins and other nutrients, such as vitamins and minerals, thereby reducing their bioavailability and potentially triggering various adverse physiological effects. Some proteins also have allergenic effects on sensitive individuals [31,32]. Furthermore, the authors mention phytoestrogens, a structurally diverse group of plant phenolics that comprises isoflavones, lignans, stilbenes, and coumestans. One of the key issues associated with ANFs is the limited bioavailability of minerals, due to the presence of anti-mineral compounds such as phytates and oxalates, which are salts of phytic and oxalic acids, respectively [33]. While categorized as ANF, under certain conditions and dosages, these bioactive compounds can exhibit beneficial roles in preventing human diseases [32] and exhibit anabolic potential comparable to animal proteins [34]. Several methods in vitro have been developed to assess protein nutritional quality. For instance, the protein digestibility-corrected amino acid score (PDCAAS) compares the amount of the first limiting IAA in a protein with the amino acid requirements of a reference population group, where the WHO considers 1.0 to represent the highest possible protein quality [35]. Additionally, the digestible indispensable amino acid score (DIAAS) is used to assess protein quality, and proteins can be categorized as: excellent quality (DIAAS ≥ 100), high quality (DIAAS 75–99), or low quality (DIAAS < 75) [10]. Both criteria provide useful information about the ability of proteins to provide enough essential amino acids to meet the nutritional requirements in humans. In this line, the DIAAS value of different plant-based sources ranges from 56.8% to 94%, reflecting limitations in lysine, methionine, cysteine, leucine, threonine, tryptophan, and/or isoleucine [22,36,37]. However, these limitations can be mitigated by combining complementary plant protein sources or fortifying plant-based foods with limiting amino acids.

Besides nutritional characteristics, challenges associated with plant-based matrices are related to technological properties for food product development and the sensory impact, particularly due to undesirable odors and flavors, e.g., beany, grassy, astringency, earthy, and bitter notes [38,39], as well as texture problems like lack of juiciness, firmness, elasticity, or creaminess [39]. For this reason, this section provides a description of the composition and limitations of the main sources of plant protein ingredients, including legumes, cereals/pseudocereals, and oilseeds, which are used as raw materials to develop plant-based ingredients and foods.

### Plant-Based Techno-Functional Ingredients

Plant-based protein sources provide a diverse range of macronutrients and bioactive compounds, making them suitable for the development of plant-based foods. Each source provides unique nutritional and techno-functional properties that distinguish it from traditional animal-derived products. However, converting these protein sources into techno-functional ingredients is a critical step. Therefore, the careful selection of raw materials (as previously described), their specific form, and the extraction process are key determinants of achieving a successful final product. Regarding their form, plant protein ingredients are commercially available in three types: (i) flours, containing 20–30% protein; (ii) concentrates, containing 50–80% protein; and (iii) isolates, containing more than 90% protein [40]. The obtention of plant-based ingredients can be achieved by protein extraction using chemical, biological, and physical methods, each offering distinct advantages for maximizing yield and quality. Chemical methods involve altering protein solubility to induce precipitation, typically by using acids, alkalis, or organic solvents. Biological methods, such as enzyme-assisted extraction with pectinase, cellulase, or glucoamylase, enable selective recovery of high-quality proteins while minimizing denaturation. Physical methods, including high-pressure treatment, microwaves, pulsed electric fields, and ultrasonic-assisted extraction, disrupt cell walls to facilitate solvent penetration and protein release [41,42]. These complementary approaches provide versatile strategies for obtaining techno-functional plant-based protein ingredients for food applications.

The most widely used plant-based ingredient in the development of alternative foods is soy protein isolates (SPI); this isolate remains a benchmark due to its high protein content (~90%) and digestibility (87.6%), coupled with versatile functional properties such as solubility, gelation, emulsification, and colloidal stability, which facilitate its extensive use in analog product development [43,44]. In addition to SPI, several alternative protein sources have gained increasing scientific attention in recent years. Among them, chickpea protein isolates have demonstrated high protein content and promising techno-functional properties such as solubility, emulsifying ability, and foaming stability, supporting their application in plant-based foods [45,46]. Lupin has also emerged as a valuable protein source due to its climate resilience and considerable protein content (25–45 g/100 g); recent studies have shown that lupin protein isolates exhibit favorable amino acid composition and functionality comparable to commercial isolates, highlighting their potential for use in food applications [47]. Additionally, proteins from other legumes, such as pea and mung beans, and pseudocereals, such as quinoa and amaranth, have been explored for their gelation capacity, emulsifying behavior, digestibility, and suitability for food alternatives. In this context, Sajib et al. [48] demonstrate that pea protein isolate, after an optimized process that controls pH and temperature, exhibits strong emulsifying and foaming capabilities and is often favored for its relatively lower allergenic potential compared to soy, thereby expanding its applicability across diverse consumer segments. In addition, more complex and less commonly used sources include mung bean protein isolate (MPI), which exhibits desirable physical properties after the texturization process, improves the fibrous structure, and shows great potential for consumption as a meat extender [49]. Additionally, MPI exhibits valuable techno-functional properties, including good solubility, high oil absorption capacity, and emulsifying and foaming abilities. Moreover, its biofunctional composition provides detoxifying and cholesterol-lowering activities, making it a promising candidate for use as a nutraceutical ingredient [49,50]. On the other hand, protein concentrates from pseudocereals like quinoa and amaranth are distinguished by their high digestibility (80–90%) and balanced amino acid profiles, alongside favorable foaming and emulsifying capacities, making it an attractive option for clean-label formulations [51]. Thus, this diversity in protein origin enables food processors to strategically combine sources to balance functionality, nutrition, and sensory characteristics in plant-based analogs.

The potential of the various protein alternatives is undeniable; however, several persistent challenges limit the widespread adoption and application of these ingredients in the development of innovative plant-based alternatives. The obstacles include the presence of ANFs, poor sensory characteristics, extended cooking times, and incomplete amino acid profiles [52]. Additionally, it is essential to recognize that transforming plant-based ingredients into products capable of properly mimicking conventional animal-derived proteins remains a significant technological challenge. For instance, understanding the relationship between starch and protein (usually found in flours of wheat, corn, peas, and beans) is essential because their interactions during thermal processing can affect gelatinization and the texture of the final products [53]. Plant protein isolates also face limitations related to their inability to assemble structural configurations that replicate the functionality of animal proteins, including desirable attributes such as appearance, texture, water and fat binding, cookability, and mouthfeel. Therefore, adequate processing is necessary to enhance nutrient bioavailability, nutritional value, and techno-functionality of food. The processing technologies should be tailored to the specific protein types in each matrix. For example, globular proteins, which can be found in proteins isolated from soy, pea, mung bean, potato, and rice, are the most used in plant-based foods due to their emulsifying, foaming, and gelling capacities [54]. These functionalities arise from their ability to unfold and aggregate through hydrophobic and disulfide interactions during heating. However, the performance of plant protein ingredients often differs from their animal counterparts due to variations in thermal denaturation temperatures and isoelectric points, as well as their degree of aggregation and native assembly [22]. Unlike flexible casein proteins or fibrous collagen, abundant in animal systems, plant proteins rarely exhibit similar structures, making their replicating structures particularly complex. Although some fibrous structures can be mimicked using gluten or specific mycoproteins, options remain limited [22,54]. Additional challenges include undesirable off-flavors (e.g., beany, grassy, earthy), poor solubility, batch-to-batch variability, and low purity due to the presence of residual seed hulls or other non-protein components.

Despite the numerous advantages of plant-based sources, critical reflection is essential to understand and address their limitations for successfully guiding the development of innovative, healthier, and more sustainable food products. In this context, the next generation of plant-based foods should focus on harnessing the full compositional complexity of raw plant materials. By applying processing strategies, it is possible to assess their techno-functional potential while addressing current formulation challenges. In this context, microorganisms with strong enzymatic activity can be utilized through fermentation to overcome the sensory, nutritional, and functional limitations of plant-based sources. Microorganisms can serve as a sustainable and versatile source of nutrients and bioactive compounds, offering novel solutions to meet evolving consumer needs. Therefore, in the next section, the food processor and researcher seek information about the effects of different microbial strategies in developing innovative food ingredients.

## 3. Microbial Strategies for Innovative Ingredient Production in Novel Food Alternatives

Microorganisms in the food industry have been, and continue to be, crucial for the development of this productive sector, serving as the basis for food transformation and preservation processes, as well as for food safety assurance strategies. Their metabolic versatility has also contributed to the creation of innovative products. More recently, research has explored the complex metabolic potential of microorganisms in producing specific compounds such as enzymes, aromatic molecules, antimicrobials, and polysaccharides that enhance the texture, flavor, and nutritional quality of fermented foods [55]. With this wide range of metabolic capabilities, it is important to recognize that the first step toward effectively using microorganisms in the plant-based protein sector is an appropriate selection at species and strain levels. However, this selection should not be based solely on the potential activity or benefits conferred to the substrate. Operational challenges must also be considered. Therefore, selecting food cultures requires a systematic, multi-step approach, including: (i) screening for tolerance to stressful fermentation conditions, particularly the complex composition of plant-based substrates; (ii) identifying strains capable of producing key metabolites and changes in the plant-based substrate, aiming to maximize functional properties while avoiding undesirable traits. and (iii) evaluating relevant technological parameters, carefully considering key operational factors such as inoculum concentration and timing of inoculation, fermentation time, and temperature to achieve the desired outcomes [56]. In addition, microbial selection must consider their safety status and regulatory acceptance. Species recognized as GRAS (Generally Recognized as Safe) or QPS (Qualified Presumption of Safety) are preferred options, as they facilitate industrial adoption and market acceptance while ensuring consumer safety. Therefore, a holistic fermentation strategy is needed, grounded in a deep understanding of microorganism–substrate interactions that allow the tailored design of fermentation processes, maximizing benefits while acknowledging the inherent advantages and limitations of microbial applications in plant-based food production. This section provides key information about the roles of traditional fermentation, biomass fermentation, and precision fermentation, with a focus on the alternative protein sector. Through this approach, it is possible to identify microorganisms suitable for industrial-scale processing.

### 3.1. Traditional Fermentation to Improve the Nutritional Quality of Plant-Based Derivatives

#### 3.1.1. Antinutritional Factors

**Figure 1:**
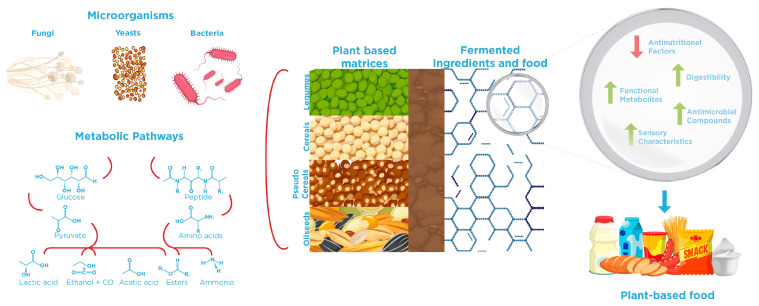
Interaction between the potential application of microorganisms and plant-based matrices to improve nutritional quality through traditional fermentation.

Understanding microorganism–substrate interactions is fundamental for the efficient fermentation processes and the development of ingredients and alternative food products (Figure 1). In this specific case, the plant-based sources are considered substrates with a complex chemical and structural composition, where ANFs could be present. Controlled fermentation with specific microbial strains is especially advantageous for improving the nutritional profile of plant-based ingredients, as evidenced by the examples presented in Table 1. In this line, Lactic Acid Bacteria (LAB) possess versatile metabolic pathways that enable the degradation of various ANFs commonly found in plant-based sources. Indeed, LABs have been extensively studied for their ability to mitigate ANFs through specific enzymes and transport systems encoded in their genomes. As reviewed by Molina et al. [57], LAB targets different classes of ANFs via specialized metabolic pathways. For example, phytates are hydrolyzed by phytases (*phy*), releasing inorganic phosphate and myo-inositol, while raffinose family oligosaccharides, such as raffinose and stachyose, are degraded by α-galactosidases (*melA*) and β-fructofuranosidases (*levS*, *fflA*), improving carbohydrate digestibility. Phenolic acids can be decarboxylated or structurally modified by phenolic acid decarboxylase (*pad*) and galactose decarboxylases (*lpdc*), thereby reducing their astringency and minimizing interference with nutrient absorption. Oxalate metabolism is facilitated by enzymes such as oxalyl-CoA transferase (Ox) and formyl-CoA transferase (Frc), which convert oxalate into less harmful metabolites. Furthermore, LAB possess proteolytic systems—*prt* (envelope proteinase), *opp* (oligopeptide transport system), and *dpp* (di-/tripeptide transport system)—capable of degrading enzyme inhibitors, including trypsin inhibitors, thereby enhancing protein bioavailability. Another important aspect that occurs during fermentation is that a decrease in pH leads to the activation of endogenous enzymes, which significantly contributes to the reduction of phytic acid in fermented plant matrices [58]. For instance, *Lactiplantibacillus plantarum* and *Pediococcus acidilactici* have been reported to reduce phytic acid, trypsin inhibitors, and total phenolic content in lentils, green peas, and pea flours [59]. Similarly, chickpea flours fermented with *Lacticaseibacillus casei* LBC491, *Lactiplantibacillus plantarum* 299v, *Leuconostoc mesenteroides* OM94, and *L. plantarum* E75 showed a significant reduction in phytic acid compared to non-fermented controls [60]. Other studies demonstrated that *Lactiplantibacillus plantarum* CRL 2211 and *Weissella paramesenteroides* CRL 2182 effectively decrease tannin content, with significantly greater reductions observed during extended fermentation periods of bean flour [61]. Co-culture strategies have also proven advantageous, leading to greater degradation of trypsin inhibitors than spontaneous fermentation. Furthermore, *L. plantarum* MRS1 and *Levilactobacillus brevis* MRS4 have been shown to simultaneously reduce phytic acid, condensed tannins, raffinose, and trypsin inhibitor activity, along with lowering the starch hydrolysis index in legume flours [62].

Filamentous fungi also play a crucial role in reducing ANF, particularly in phytate degradation. *Penicillium*, *Aspergillus*, *Mucor*, and *Rhizopus* genera are recognized for their high phytase productivity. These enzymes function as biocatalysts in the partial or complete hydrolysis of phytic acid to myo-inositol phosphates and inorganic phosphate. This bioconversion not only reduces the antinutritional properties of phytates but also prevents the formation of enzyme–protein complexes and the chelation of nutritionally important metal ions [63]. For example, the fermentation of fava bean flour by *Aspergillus oryzae* and *Rhizopus oligosporus* decreased phytic acid, tannins, and chymotrypsin inhibitors, while *Aspergillus oryzae* completely eliminated saponins and *Rhizopus oligosporus* reduced them by 19.4% [64]. Also, fermentation of the tuberous legume marama beans with *A. oryzae* and *Aspergillus sojae* significantly lowered phytic acid and trypsin inhibitor levels compared to non-fermented flours [65]. The use of *Pleurotus ostreatus* has shown more pronounced degradation of phytate contents in flours than in whole grains. For lentils, reductions reached 27% in grains and 89% in flour; in quinoa, 45% in seeds and 90% in flour. These changes became significant after the 10th day of fermentation, depending on substrate characteristics [66]. On the other hand, the fermentation system itself is also critical. For example, a two-step solid-state fermentation (SSF) starting with *Bacillus subtilis* followed by *A. oryzae* achieved greater reductions in phytic acid, total tannins, and saponins than fermentation with *A. oryzae* alone, demonstrating that sequential inoculation increases the efficiency of ANF removal in soybean meal [67].

#### 3.1.2. Digestibility

Microorganisms have a broad capacity to enhance digestibility, and the extent of this effect depends on the microbial strain, fermentation time, and type of fermentation. During fermentation, proteolysis occurs, breaking down complex protein structures into simpler peptides and amino acids. This process can improve the digestibility of plant-based ingredients, making fermentation one of the most effective strategies to enhance protein bioaccessibility from plant-derived foodstuffs. To estimate the effect of fermentation on protein digestibility, in vitro protein digestibility (IVPD) is usually applied [36]. For example, Cabuk et al. [68] demonstrated that the IVPD of pea protein concentrate increased as fermentation time progressed, reaching 87% after 5 h using *L. plantarum* NRRLB-4496 in submerged fermentation (SmF). A similar time-dependent relationship has been reported for underutilized raw materials such as cassava flour, where Anyiam et al. [69] observed a maximum IVPD of 62.42% in fermented cassava compared to the unfermented sample after 42 h of spontaneous fermentation. In this line, spontaneous fermentation of locust bean flour, a novel potential source of high-quality protein, resulted in a higher IVPD of 74.29% compared to the unfermented matrix (66.47%) after 72 h of process [70]. Whereas, in the production of traditional fermented foods such as koji and tempeh using rice as a substrate in SSF with *A. oryzae* and *R. oligosporus*, no significant changes in IVPD values were observed compared to the unfermented control, maintaining values close to 80.9% [71]. In addition, fermentation of pea protein concentrates with *L. plantarum* increased IVPD from 70% to 87% after 5 h, while reducing protease inhibitor activity. However, prolonged fermentation with the *L. plantarum* strain, which diminished sulfur amino acids, resulted in a decrease in vitro PDCAAS [68]. Under certain conditions, other negative impacts of fermentation on digestibility have been reported. In this context, Stone et al. [72] documented a reduction in the IVPD of chickpea, lentil, and faba bean protein isolates, regardless of the microorganism used (*A. oryzae*, *A. niger*, or *L. plantarum*), with decreases ranging from moderate (80–82%) to pronounced (74–77%) compared to initial values above 87%. A similar situation was reported by Zwinkels et al. [71], during *koji* and *tempeh* production using barley as the raw material, where IVPD values dropped to 86–87% after fermentation with *A. oryzae* and *R. oligosporus*, respectively, compared to 90–91% in the unfermented control. These findings suggest the complexity of plant-based matrices, together with the multitude of biochemical interactions involved. One proposed explanation relates to the increase in phenolic compounds during fermentation, which can interfere with protease activity on protein substrates and inhibit enzymatic hydrolysis [73]. Another hypothesis suggests that during fermentation, conformational changes occur in protein structure; for example, an increased proportion of β-sheets in fungal proteins has been associated with reduced IVPD [71]. Thus, it is necessary to consider that during fungal fermentations, they can generate 6–13% fungal biomass, whereas bacterial fermentations typically yield around 0.5% biomass [74]. This information suggests that microbial biomass accumulation must be considered during the production of fermented plant-based ingredients.

Similarly, raffinose family oligosaccharides, including raffinose, stachyose, and verbascose, are α-galactosyl derivatives of sucrose found abundantly in legumes. Although they are not directly toxic, these carbohydrates are poorly digested in the human gastrointestinal tract and are fermented by gut microbiota, often leading to gas production and flatulence [57]. Therefore, mitigating these compounds through bioprocessing, particularly through fermentation, represents a relevant step in the development of high-quality plant-based protein ingredients or new food products.

#### 3.1.3. Functional Metabolites

The proteolytic action of certain microorganisms can release bioactive peptides that impart unique characteristics to foods. Bioactive peptides are specific protein fragments (typically 2–20 amino acids) that exhibit various biological activities, including antioxidant, antihypertensive, anticancer, and anti-inflammatory properties, and have been associated with health effects [75]. Though the release of these compounds during the development of plant-based ingredients or products can be achieved through chemical, technological or biological processes, the latter, mainly fermentation, offers additional benefits that are not typically found in other processes. This advantage is primarily due to the diversity of proteases that can be activated during fermentation, resulting in the generation of peptides with different sizes and sequences, and consequently, a broader spectrum of biological activities [75,76]. When considering the production of bioactive peptides, the fermentation system employed plays a crucial role. For instance, SSF favors the production of ingredients or final products with enhanced functional characteristics, as it promotes microbial growth and the generation of specific peptides within the same matrix. In contrast, SmF facilitates the release of bioactive peptides into the aqueous phase, requiring subsequent downstream operations (e.g., centrifugation, filtration) for their recovery. Importantly, both fermentation systems allow the use of different microorganisms and substrates. A study by Limon et al. [77] demonstrated that the combination of fermentation system and microbial strain influences the properties of bioactive peptides produced from kidney bean flour. Specifically, extracts obtained from SSF using *Bacillus subtilis* exhibited higher levels of antioxidant activity, whereas extracts from SmF with *L. plantarum* displayed notable antihypertensive potential. Classic plant-based ingredients or foods known to be rich sources of bioactive compounds with health-promoting effects include soy-derived products such as tempeh, natto, and miso, all traditionally produced via SSF. In line with this, Harahap et al. [78] reported that enzymes such as protease, leucine aminopeptidase, carboxypeptidase, glutaminase, γ-glutamyl transferase, and amylase, along with microbial strains like *Rhizopus* spp., play a crucial role in transforming complex soy nutrients into bioavailable forms. Similarly, Ayyash et al. [79] describe how the antioxidant activity observed in quinoa fermented by *Limosilactobacillus reuteri* and *L. plantarum* after 24 h can be attributed to the proteolytic products released during the fermentation process. Quinoa proteins, which are rich in lysine, generate short-chain peptides (<9.0 kDa) with notable antioxidant capacity. Regarding antihypertensive activity, the higher ACE-inhibitory effects of lupin and quinoa fermented with *L. reuteri* and *L. plantarum* K779 have been linked to functional proteolytic systems that produce bioactive peptides with targeted antihypertensive properties [79].

**Table 1:** Microbial species employed in traditional fermentation to enhance the nutritional quality of diverse plant-based derivatives.

Microorganisms	Plant-Based Ingredients	Fermentation Conditions	Effect	References
*Lacticaseibacillus casei* LBC491, *Lactiplantibacillus plantarum* 299V, *Leuconostoc mesenteroides* OM94, *L. plantarum* E75	Chickpea flour	SmF, 24 h, 30–37 °C	Reduce phytic acid	[60]
*L. plantarum* CRL 2211, *Weissella paramesenteroides* CRL 2182	Bean flour	SmF, 24 h, 37 °C	Decrease tannin content	[61]
*L. plantarum* MRS1, *Levilactobacillus brevis* MRS4	Legume flours	SmF, 24 h, 30 °C	Reduce phytic acid, condensed tannins, raffinose, trypsin inhibitors, and starch hydrolysis index	[62]
*Aspergillus oryzae* *Rhizopus oligosporus*	Faba bean flour	SSF, 24–72 h, 28–30 °C	Decrease phytic acid, tannins, chymotrypsin inhibitors, saponins (*A. oryzae* eliminated completely)	[64]
*Aspergillus oryzae*, *Aspergillus sojae*	Marama bean flour	SSF, 8 days, 30 °C	Reduce phytic acid, trypsin inhibitors	[65]
*Pleurotus ostreatus*	Lentils and Quinoa flours	SSF, 14 days, 25 °C	Increased protein and reduced phytate content.	[66]
*Bacillus subtilis* + *A. oryzae* (sequential inoculation)	Soybean meals	two-step SSF, 96 h 25 °C,	Reductions in phytic acid, tannin, and saponins	[67]
*L. plantarum* NRRLB-4496	Pea protein concentrate	SmF, 32 °C, 11 h	Protein digestibility increased to 87.4%	[68]
*A. oryzae*, *R. oligosporus*	Rice	SSF; *A. oryzae* 32 °C, 68 h, *R. oligosporus* 28 °C, 44 h	No significant change In Vitro Protein Digestibility (IVPD)	[71]
*A. oryzae*, *A. niger*, *L. plantarum*	Chickpea, Lentil, Faba bean	SSF, 30–37 °C, 48 h	Reduction IVPD (from 87% to 74–82%)	[72]
*Bacillus subtilis*	Kidney beans extracts	SSF, 48 h	Higher contents of soluble phenolic compounds and antioxidant activity	[77]
*L. plantarum*	Kidney beans extracts	SmF, 96 h	Antihypertensive peptides	[77]
*Rhizopus* spp.	Soybean meals	SSF, 24–72 h, 25–37 °C,	Increase digestibility, release of bioactive compounds	[78]
*L. reuteri*, *L. plantarum*	Quinoa, Lupin, Wheat flours	SSF, 72 h	Antihypertensive activity, the higher ACE-inhibitory effects of lupin and quinoa	[79]

During fermentation, various classes of phenolic compounds are transformed into derivatives that often exhibit greater bioactivity than their original forms. This process commonly results in enhanced total phenolic content and antioxidant capacity, depending on the microbial strain and the duration of the fermentation, as reported in several studies [80,81,82]. Specifically, *L. plantarum*-based fermentation for 72 h improved the phenolic composition and strengthened the antioxidant and anti-hyperglycemic properties of chickpea-derived ingredients, contributing to their development as functional ingredients with enhanced nutraceutical potential [83]. The fermentation of quinoa seeds with *R. oligosporus* and *A. oryzae* resulted in a significant increase in total phenolic content (TPC) and total flavonoid content (TFC) over time, indicating progressive enrichment of bioactive compounds during the fermentation process [84,85]. Likewise, *L. plantarum* T6B10 and *Furfurilactobacillus rossiae (Lactobacillus rossiae* T0A16) fermentations of quinoa flour resulted in substantial increases in TPC, with fermented quinoa pasta and sourdough exhibiting significantly higher phenolic concentrations compared to their non-fermented counterparts [86]. Thus, TF is a promising approach to improve the phenolic profile and functional quality of plant-based ingredients, thereby enhancing their potential for use in health-promoting and functional food applications. Nonetheless, the biotransformation of certain phenolic subclasses remains underexplored, primarily due to their low concentrations and perceived limited impact on the overall food matrix. This highlights an opportunity for the application of metabolic engineering strategies to further boost the levels and bioactivity of specific phenolic compounds in fermented plant-based ingredients and foods.

Based on the abovementioned information regarding the generation of bioactive peptides or phenolic compounds, it is useful to consider the intrinsic composition of different plant-based groups. Legumes emerge as the primary candidates for peptide production owing to their high protein content and balanced amino acid profile, while also providing a relevant pool of isoflavones and flavonoids. Pseudocereals, with their nutritionally superior proteins and diverse phenolic spectrum, also could represent a dual-purpose source. Oilseeds, although traditionally valued for their lipid fraction, also contribute to the production of bioactive peptides and distinctive phenolics, such as lignans, with relevance for antioxidant applications. Finally, cereals, especially their bran fractions, stand out as abundant reservoirs of phenolic acids, with more limited protein availability for peptide generation. Based on these characteristics, a proposed hierarchy can be established: legumes > pseudocereals > oilseeds > cereals for peptides, and cereals/oilseeds > legumes/pseudocereals for phenolics. This classification can serve as a practical guide when selecting raw materials for fermentation purposes in the design of functional ingredients or nutraceutical development, based on plant sources.

#### 3.1.4. Antimicrobial Compounds

Fermentation is a useful technology for producing compounds that inhibit spoilage and pathogenic microorganisms. Traditionally, fermentation has been used to ensure food safety and extend the shelf life of perishable foods, such as meat, milk, and vegetables, while improving their organoleptic properties and nutritional value. Lactic acid fermentation is a widely used type of fermentation in which LAB can produce different bioactive compounds with antimicrobial activity, such as organic acids (lactic, acetic, propionic, etc.), diacetyl, ethanol, hydrogen peroxide, CO_2_, and bacteriocins [87]. The potential of the LAB as a green and sustainable strategy for food safety and preservation has attracted researchers and industry, and bacteriocinogenic or bioprotective cultures are increasingly being used by the food industry due to their efficacy and “clean label” consideration [88]. This antimicrobial efficacy has been demonstrated in fermented soybean meal and other plant matrices. For example, *L. plantarum* P15 and *Enterococcus faecalis* ZZUPF95 inhibit the growth of *Staphylococcus aureus*, *B. subtilis*, *Escherichia coli*, *Listeria monocytogenes*, *Pseudomonas aeruginosa*, and *Micrococcus luteus*, through bacteriocin production and acidification in fermented soybean meal [89]. The combined process (fermentation and enzymatic) also has a positive effect on the fermentation process of soybean meal by *E. faecalis* ZZUPF95 and acid protease, leading to a decrease in pH and, consequently, the control of coliform and aerobic bacteria [90]. Additionally, the fermentation of faba bean flour with *L. brevis* AM7, which produces antimicrobial peptides, has demonstrated the broadest inhibitory spectrum against mold contamination related to *Penicillium*, *Eurotium*, and *Aspergillus* species, a significant concern in the food industry [91]. Bacillus species have also demonstrated significant potential in the development of plant-based ingredients with antimicrobial properties. For instance, *Bacillus* spp. strain LM7, used in the production of fermented soybean paste, has been reported to produce antimicrobial lipopeptides belonging to the surfactin and bacillomycin families that exhibited inhibitory activity against pathogenic bacteria such as *B. cereus*, *L. monocytogenes*, and *E. faecalis*, as well as fungal and yeast strains including *Saccharomyces*, *Aspergillus*, and *Fusarium* [92]. In the same line, hydrolysates from fermented soybean food (natto) have been shown to contain antimicrobial peptides effective against *Streptococcus pneumoniae* R6 and several *Bacillus* species (*B. subtilis*, *B. pumilus*, *B. licheniformis*, *B. cereus*, and *B. megaterium*) [93]. On the other hand, the fermentation of agri-food by-products, some of which already contain compounds with antimicrobial activity, such as phenolic compounds [94], is another interesting strategy in the context of the circular economy for obtaining antimicrobials. In this regard, the antimicrobial activity of tomato, melon, and carrot by-product extracts showed in vitro and in foodstuff activity against spoilage microorganisms and foodborne pathogens such as *Salmonella* spp., *L. monocytogenes*, and *B. cereus* [95].

#### 3.1.5. Sensory Characteristics

From a sensory perspective, TF has proven to be an effective strategy for mitigating undesirable odors in plant-based proteins and by-products by either decomposing or masking off-flavors and generating pleasant aroma-active metabolites [96,97]. In this context, Shi et al. [96] reported that lactic acid fermentation effectively improved the overall quality of pea protein isolates in terms of appearance, aroma, and flavor. This effect was achieved by *L. plantarum* after 10 h of fermentation. The effect of LAB (e.g., *L. plantarum*, *L. parabuchneri*, *L. brevis*, and *Lactobacillus helveticus*) has been shown to significantly reduce green and beany notes in lupin and pea protein isolates by lowering the concentration of aldehydes such as n-hexanal, while simultaneously enhancing floral and honey-like aromas [98,99]. Similarly, yeast fermentation of okara can transform grassy off-flavors into fruity and sweet notes through the enzymatic conversion of aldehydes into alcohols, acids, and esters [100]. Moreover, the combination of LAB and yeasts in pea protein-based beverages enhances aroma quality by degrading off-flavor compounds and producing fruity and floral esters [101]. Certainly, TF is a promising strategy for enhancing the sensory quality of plant-based proteins, but its effectiveness depends strongly on the strains and conditions used. Although it shows great potential, further research is needed to optimize processes and fully harness its benefits for novel food development. To illustrate the mechanisms of the sensory effects described above, Table 2 summarizes the main flavor-active metabolites produced by microorganisms discussed in this review, highlighting their metabolic origins, chemical classes, and associated aroma descriptors.

On the other hand, TF can substantially modify the techno-functional properties of alternative food ingredients. Controlled microbial fermentation can alter solubility, gelling ability, water absorption, and emulsifying properties of proteins, depending on the substrate and the microorganisms involved. For instance, SSF with *P. ostreatus* has been shown to enhance protein solubility in oat flour and mixed *Chlorella vulgaris–oat* flour systems, as well as to improve the gelling and water absorption capacities of quinoa, the oil absorption capacity of chickpea and oat, and the emulsifying properties of chickpea [24]. Similarly, *A. oryzae*-fermented quinoa flour exhibits increased water- and oil-holding capacities; however, its foaming and emulsifying properties are lower compared to the non-fermented material [109]. In the case of lupin flour, SSF with *A. sojae* and *A. ficuum* increased swelling capacity to 3.28% and 2.09%, respectively, and to 2.24% in co-culture fermentations, compared with 1.54% in the non-fermented control, although a reduction in water absorption was also observed during fermentation [110]. In addition, complementary strategies have been implemented to further improve techno-functional properties. For example, bioprocessing sorghum and cowpea flours with lactic acid bacteria and specific amylases has led to measurable increases in protein solubility, pasting viscosity, and water-binding capacity [111]. Despite many studies reporting statistically significant differences in techno-functional properties after fermentation, these improvements are not always sufficient to meet targeted textural requirements when fermented ingredients are used alone. Therefore, combining fermentation with complementary processing technologies, such as high- or low-moisture extrusion, protein blending, and targeted enzymatic treatments, has emerged as a promising approach to obtaining ingredients with improved functional performance and meat- or matrix-like textures [112,113].

Overall, TF remains a powerful tool for tailoring the nutritional, sensory, and functional traits of alternative ingredients. However, its practical application in product development typically requires multi-step processing chains, in which fermentation acts as an essential component among extrusion, formulation, and post-processing stages.

### 3.2. Biomass Fermentation: Microorganisms as a Source of Alternative Proteins

Microorganisms have several advantages, including their metabolic versatility and capacity to address pressing challenges, such as providing scalable protein at low cost and with minimal environmental impact through microbial protein production. There is growing interest in the use of microbial protein, also known as single-cell protein (SCP), bioprotein, or biomass, to meet the global demand for nutritious food, as it has various advantages over conventional plant sources. These benefits include nutritional composition, circular processes, short production times (minutes to hours) with no extensive land use. SCP is an edible unicellular microorganism derived from several species of microorganisms, primarily fungi, microalgae, yeasts, or bacteria [114]. These groups of microorganisms show distinct nutritional compositions on a dry weight basis. Fungi contain 30–45% protein, 2–8% fat, 9–14% ash, and 7–10% nucleic acids, while macroalgae are richer in protein (40–60%) and fat (7–20%), with 8–10% ash and a lower nucleic acid content (3–8%). Yeasts provide 45–55% protein, 2–6% fat, 5–10% ash, and 6–12% nucleic acids, representing a balanced nutrient profile. Bacteria stand out as the richest in protein (50–65%) but have very low fat (1–3%), 3–7% ash, and relatively high nucleic acid levels (8–12%) [115,116]. In general, SCP has a complete amino acid profile, including lysine, methionine, and threonine, which satisfies nutritional requirements. However, it can be deficient in sulfur-containing amino acids such as methionine and cysteine [117]. Whereas high levels of minerals, enzymes, and vitamins from group B (e.g., riboflavin, thiamine, pyridoxine, cobalamin, among others) have been observed [118]. In addition to their nutritional value, SCP has an important presence of biologically active compounds that confer several biofunctional properties. For instance, algae SCP contains a range of peptides associated with antioxidant, anti-proliferative, anti-inflammatory, antihypertensive, anti-diabetic, and antimicrobial properties [119]. Furthermore, fungal proteins exhibit a range of functional properties, including regulating lipid and cholesterol levels, increasing satiety, promoting digestion, improving immunity, and facilitating intestinal health [120]. Therefore, this remarkable complexity makes SCP highly versatile for a wide range of applications, from food to feed production. However, the main concern in SCP is the presence of a high concentration of nucleic acids derived from DNA and RNA of microbial cells, mainly derived from RNA due to rapid microbial growth. After consumption, purines from nucleic acids are metabolized into uric acid, whose accumulation in the serum is associated with the formation of kidney stones and gout [121]. Among the methods to minimize nucleic acid content of SCP, heat treatment (i.e., at 60–75 °C for several minutes) can be applied to activate endogenous RNases to break down ribonucleotides that diffuse out of the cells and are subsequently removed by centrifugation or filtration, minimizing the loss of protein quantity and quality [122,123]. Other types of compounds that may develop during microbial growth, such as mycotoxins and cyanotoxins [124], represent significant health concerns. Therefore, the careful selection of microorganisms and the conditions for biomass production are crucial when initiating an SCP production process, as they must meet specific quality criteria. These include high nutritional value (adequate protein content and bioavailability), safety (absence of toxins and low nucleic acid content), consumer acceptance (desirable taste, flavor, and texture), and cost-effectiveness in production [125].

#### 3.2.1. Fermentation Methods: Submerged and Solid State

The production of SCP involves several stages beyond the core fermentation process. From a technological perspective, it can be achieved through two main approaches: SmF and SSF. Each method presents specific advantages and limitations, ranging from process economics to the ability to control key operational parameters. Both types of fermentation process include upstream operations such as substrate pre-treatment, media formulation, inoculum preparation, and the fermentation conditions, as well as downstream steps like microbial biomass harvesting, post-treatments (purification, concentration, etc.), and the final adequacy of SCP (stabilization, formulation) for food or dietary supplement applications [126]. Additional considerations include process scalability and costs, as well as the integration of circular bioeconomy principles. SmF is generally regarded as the most advantageous process, as it enables superior control over fermentation parameters, facilitates downstream separation, and offers the potential to produce multiple value-added products. On the other hand, SSF provides a valuable opportunity for the valorization of diverse substrates, including various waste/streams, aligned with circularity, which allows the utilization of low water usage and energy consumption. Nevertheless, SSF presents significant challenges, including limited process control and, most critically, the difficulty of recovering SCP, since the microorganisms grow and adhere to the solid substrate [23,127].

#### 3.2.2. Substrates and Microorganisms

**Figure 2:**
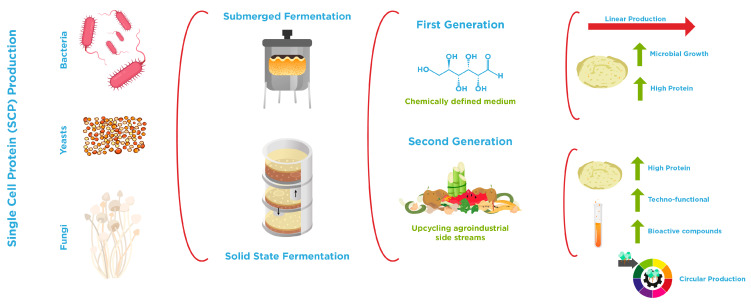
Schematic overview of first- and second-generation single-cell protein production using biomass fermentation.

Microorganisms can grow on a wide variety of substrates, ranging from simple (chemically defined media) to more complex ones that incorporate by-products or waste streams (Figure 2). In this context, Li et al. [115] proposed a classification of SCP into first- and second-generation fermentation products. First-generation SCP primarily involves the use of microorganisms to convert organic substrates (rich in glucose) into biomass, cultivated under controlled conditions to optimize growth and protein production. The most common microorganisms associated with this approach belong to the genera *Fusarium* and *Saccharomyces* (conventional yeast), which can be utilized in various food products and supplements. In contrast, second-generation SCP explores more innovative strategies, such as upcycling agricultural by-products or waste into protein-rich biomass and producing functional metabolites (including organic acids and bioactive peptides). This approach also encompasses the use of genetically modified microorganisms (discussed in Section 3.3) and advanced bioreactor designs to enhance protein yields while reducing production costs. For instance, *Methylobacterium organophilum* growth in methanol (carbon source) showed a high percentage of crude protein (54.1%) and the presence of almost all essential amino acids, suggesting the possible use of this microbial biomass as a SCP, especially for animal feed formulations. In addition, *M. organophilum* can produce carotenoids, making this bioprocess commercially viable [128]. *Yarrowia lipolytica* (non-conventional yeast) converts food waste into SCP with a protein content of 38.8 ± 0.2% *w*/*w* in biomass, with an additional chemical oxygen removal rate of 85.5 ± 0.7% [129].

#### 3.2.3. Applications and Sustainability

On the other hand, from a theoretical perspective, the potential environmental advantages of SCP production have been extensively reported and discussed by researchers. However, these claims regarding the sustainability of SCP production, particularly when by-products are employed as substrates, can only be validated through objective methodologies such as Life Cycle Assessment (LCA), which enable a comprehensive evaluation of environmental impacts. Within this framework, several stages of the SCP production chain have been identified as critical hotspots that require further investigation to ensure sustainable production. In this context, medium preparation, particularly the use of inorganic nutrients such as KH_2_PO_4_, (NH_4_)_2_HPO_4_, and MgSO_4_·7H_2_O, can account for up to 20% of climate change impacts, 50% of marine eutrophication and acidification, and 30% of water consumption, with some contributions rising to 67% in marine ecotoxicity and 71% in freshwater ecotoxicity [130,131]. The fermentation itself is another major contributor, particularly due to electricity demand for mixing, aeration, and cooling, which represent up to 68% of greenhouse gas emissions and 66% of fossil energy use, while cooling water requirements account for 55% of total water consumption [130]. In contrast, the environmental burden of downstream processing is more variable: in some cases, centrifugation and evaporation represent approximately 15% of total carbon emissions, whereas in others, centrifugation and drying were found to significantly increase the overall impacts [132,133]. Furthermore, the type of substrate and its pre-treatment have a strong influence on the environmental footprint, while inconsistencies in the definition of system boundaries in life cycle assessments of waste-derived substrates remain a source of uncertainty. Finally, evaluating SCP not only in terms of production impacts but also through its amino acid profile and nutritional quality has been highlighted as an essential challenge for positioning SCP against plant- and animal-based proteins.

Regarding the appropriate applications of SCP, based on its composition, its primary goal is to serve as an alternative protein source and represent a promising option to conventional meat products. In this line, mycoprotein stands out as a specialized form of SCP developed specifically for human consumption [134]. Mycoprotein is considered a refined subset of SCP, referring to protein-rich products derived from fungal mycelium through specialized processing techniques. Indeed, mycoprotein mimics the anisotropic structure of muscle fibers thanks to mycelium, which is the root-like superstructure of fungi, composed of individual fibrous hyphae [135]. In addition, this structure also includes protein and nutrient levels, as well as their potential to be modified and texturized to resemble meat. Among the fungal genera applied in the industrial production of mycoprotein, *Fusarium* is considered one of the most relevant. The species *Fusarium venenatum* has been widely exploited for large-scale cultivation, leading to the development of the commercial brand Quorn by Marlow Foods in the United Kingdom, which was authorized for sale as early as 1985 [136,137]. More recently, 3F BIO (Glasgow, UK) introduced ABUNDA mycoprotein, also derived from *F. venenatum*, but utilizing alternative feedstocks and cultivation processes [138,139]. In addition, Nature’s Fynd (Chicago, IL, USA) utilizes *Fusarium flavolapis* (initially deposited as *Fusarium oxysporum* MK7 and *Fusarium novum yellowstonensis*) as the basis for its mycoprotein production [140,141,142]. Additionally, their sustainable production enables these mycoproteins to exhibit a highly favorable nutritional profile, providing substantial amounts of protein (45–54 g/100 g dry weight) and dietary fiber (25–31 g/100 g dry weight), while maintaining low fat levels (4.7–13 g/100 g dry weight). They are also rich in essential micronutrients, including calcium, magnesium, phosphorus, potassium, and zinc, as well as B-group vitamins such as thiamine and pantothenic acid [135]. In addition to *Fusarium*-based mycoproteins, a wide range of microorganisms, including algae, yeasts, bacteria, and filamentous fungi, are being exploited as alternative SCP sources (Table 3). These systems employ diverse metabolic strategies, from heterotrophic fermentation to photoautotrophic cultivation, and collectively contribute to sustainable protein production with favorable nutritional profiles and reduced environmental footprints [143,144,145,146,147,148,149,150,151,152,153,154,155]. Compared to many plant- and animal-derived proteins, these SCPs combine high-quality protein content with beneficial fibers and low sodium concentrations, contributing to improved lipid profiles, energy regulation, and overall metabolic health. This balanced nutritional composition reinforces the role of SCP as a valuable contributor to both human health and sustainable food systems.

A complementary route to using microorganisms as a source of alternative protein is SSF, which takes advantage of the growth of microorganisms to transform solid matrices (plants/waste/by-products). In this context, filamentous fungi are especially attractive here because they grow vigorously on low-moisture substrates and develop extensive mycelial networks that restructure the matrix. For instance, *R. oligosporus* and *R. oryzae* have a long history in tempeh production and are exemplary SSF workhorses. Their hyphae physically entangle substrate particles, knitting them into coherent, sliceable slabs with meat-like bite and mouthfeel [156]. *A. oryzae* offers a complementary mechanism: it secretes a rich suite of proteases and related enzymes that enhance digestibility and can penetrate deeply into particles, extensively modifying internal structures, an advantage for recalcitrant inputs such as cereal brans or lignocellulosic residues [157]. In parallel, targeting organoleptic characteristics like color can be achieved by leveraging microbes that produce carotenoids, providing stable red–orange hues conducive to visually convincing red-meat analogs [39]. With a technological context, it can be inferred that the choice of the fermentation system (SmF or SSF) is crucial, depending on the intended application. For instance, SmF processes are typically used for *Fusarium* mycoprotein, whereas SSF (with *Rhizopus* or *A. oryzae*) emphasizes texture formation through hyphal entanglement and in situ enzymatic remodeling of whole particles. This information suggests SSF is well-suited to upcycling agro-industrial side streams while still delivering coherent, meat-like structures. In practice, the two strategies are complementary: *Fusarium* offers high-purity fungal biomass with a consistent filamentous texture, whereas SSF platforms can directly tune texture, digestibility, and color within complex plant matrices. Integrating these approaches can broaden the design space for next-generation, clean-label meat analogs.

#### 3.2.4. Consumer Perception and Acceptance

From the consumer’s perspective, the adoption and acceptance of SCP represent a significant challenge in advancing sustainable processes for the development of new alternative foods. In the case of mycoproteins, several factors influence consumer acceptance and purchase intentions. A European pilot study in the UK, Germany, and Romania reported that individuals often associated fungal raw materials with negative perceptions, as fungi are commonly linked to spoilage and health risk [158]. The same study highlighted that consumers expressed doubts about the healthiness and naturalness of traditional protein sources and that participants who tasted fungal protein-based meat substitutes were generally dissatisfied with the sensory qualities. By contrast, the cross-national survey conducted by Dean et al. [159] revealed a positive consumer disposition toward mycoprotein. In their study, flexitarian and vegetarian respondents reported high willingness to try and purchase mycoprotein products, motivated primarily by perceived health, safety, and environmental benefits. These findings suggest that among consumer groups already oriented toward reduced meat consumption or alternative protein adoption, acceptance of mycoprotein may be less constrained by the sensory concerns and naturalness-related skepticism. The acceptance of other SCP sources has also been investigated. For instance, UK consumers recognized microalgae as a novel food source and reported a willingness to consume algae-based products due to their perceived nutritional benefits [160]. Conversely, the willingness to try bacterial protein is significantly lower than algae and fungi, although bacterial proteins were perceived similarly to other SCP [161].

#### 3.2.5. Opportunities and Challenges

As discussed, SCP production presents numerous opportunities to enhance food sustainability and address the growing global demand for high-quality proteins. However, several critical challenges must still be overcome to fully realize its potential. These challenges range from ensuring adequate nutritional profiles to developing strategies that foster consumer acceptance, which are essential for scaling up SCP as a mainstream protein alternative. In this context, sustainability remains the primary reason for SCP acceptance, while perceived benefits can facilitate or hinder adoption depending on the extent to which consumers value these attributes and how effectively such benefits are communicated. Nevertheless, many consumers remain poorly informed about SCP, including its nutritional advantages and safety. Therefore, marketing and communication strategies should emphasize practical information, such as recipes and examples of culinary use, along with clear messages about the health and sustainability benefits, to improve consumer confidence and facilitate the integration of these products into daily diets.

Another important limitation lies in the lack of harmonized regulatory frameworks across different organizations and countries, which creates uncertainty and hinders the widespread adoption of these innovative ingredients. In addition, from a circular economic perspective, it is crucial to implement processes that maximize the efficient use of resources employed in SCP production. For instance, process streams such as fermentation supernatants (often rich in metabolites) should be explored for value-added applications, including the production of organic acids, enzymatic extracts, and other bioproducts. Ideally, these applications should be feasible without the need for extensive downstream purification steps, as additional processing would increase costs and reduce competitiveness compared to other alternative protein sources. Therefore, advancing research in these directions could contribute to building a more resilient, sustainable, and accessible SCP supply chain and should therefore be considered a priority for future investigations.

### 3.3. Precision Fermentation

Precision fermentation is a biotechnological process that uses specially engineered microbial hosts as cellular factories to produce high-value molecules, including nutritional, pharmaceutical, industrial, and chemical compounds, with high purity, consistency, and efficiency. Unlike conventional agricultural, animal-based, or chemical production methods, this approach enables the controlled synthesis of compounds that are often difficult or costly to obtain through traditional practices, thereby contributing to the development of sustainable and resilient systems. Within this framework, microorganisms have emerged as a valuable resource for enhancing the quality and functionality of plant-based foods and serving as a nutritional source of SCP in novel dietary alternatives. Their versatility lies in their metabolic diversity, which enables the production of proteins, lipids, carbohydrates, and bioactive metabolites that improve the nutritional value, texture, and sensory properties of alternative protein products. In addition, advances in the detailed understanding of the genetic and metabolic information of bacteria, yeasts, algae, and filamentous fungi have enabled the development of tailor-made organisms through synthetic biology and gene editing tools. These strategies allow the targeted production of specific molecules, the optimization of metabolic pathways, and the improvement of large-scale fermentation efficiency. Recently, Sturne et al. [162] linked the application of genetic tools to the concept of PF, emphasizing its role in the heterologous production of non-native target molecules through advanced genetic tools. The term is generally applied to two main production strategies. First, PF enables the synthesis of animal-derived proteins, such as collagen, milk proteins, egg proteins, and enzymes, through animal-free microbial systems, thereby enhancing food safety and sustainability. Second, the application of metabolic engineering tools enables the production of various ingredients, including proteins, pigments, vitamins, and fats, to upgrade the quality of plant-based alternatives [113]. In this context, despite the advantages of TF in improving quality, certain microorganisms can also generate acids such as lactic acid, ammonium, and hexanoic acid, which impart undesirable, strong, sour, or alcoholic notes [97]. This underscores the importance of PF in designing target flavor-improving strategies through the controlled microbial production of distinct molecules such as alcohols, esters, aliphatic acids, aldehydes, lactones, ketones, and other volatile compounds [163,164]. For example, *Lactococcus lactis* has been engineered to synthesize the butter-flavor compound butanedione (diacetyl) from dairy waste [165]. When integrated with lipid-based strategies, this approach can substantially enhance the sensory attributes of plant-based or alternative butter products, while also demonstrating how waste valorization can be coupled with sensory improvement. Similarly, the production of soy leghemoglobin in *Komagataella phaffii* (formerly *Pichia pastoris*) has been successfully applied to confer a meat-like flavor and color to plant-based protein meat analogs, representing one of the most established applications of PF in flavor enhancement [166].

The application of PF is a clear shift from purely research interest toward increasingly concrete and diversified applications in the alternative protein sector. This trend is further supported by recent research articles that converge on the view that PF is not merely an alternative manufacturing route but a platform technology that addresses distinct scientific, sensory, and regulatory bottlenecks that have historically limited the uptake of alternative food ingredients. Hilgendorf et al. [167] established the conceptual framework for PF as a tool to improve food quality, flavor, safety, and sustainability by metabolically editing GRAS microorganisms and utilizing abundant, low-cost feedstocks. The authors emphasize not only the ability to rewire biosynthetic pathways to generate targeted nutritional and sensory molecules but also the practical constraints that arise at the intersection of strain design and process engineering, notably, the need to close the gap between laboratory-scale pathway demonstrations and industrially relevant titers, productivities, and yields, and to adapt downstream schemes to novel product matrices. On the other hand, Eastham et al. [168] focus on the specific and critical challenge of food proteins. Their review details how designer microbes can produce animal-analog proteins and highlights bioprocess considerations that determine ingredient functionality: host selection and expression systems, medium composition and cost, folding and post-translational modifications for functionality (e.g., solubility, gelation, and emulsification), and purification strategies that preserve techno-functional properties while remaining economically viable. They also emphasize regulatory and market realities. Several protein products have already entered the commercial market, but broad adoption will require a robust demonstration of equivalence (or superiority) in functionality, as well as regulatory clearance across various jurisdictions. In another context, the discussion is also extended to SCP technologies, situating PF within a circular bioeconomy, which underscores SCP as a scalable route to biomass-based protein, drawing attention to advances in metabolic engineering (including CRISPR-based tools), utilization of second-generation feedstocks (agro-industrial residues, C1 substrates, and CO_2_-derived intermediates), and strategies to improve metabolic efficiency [115]. At the same time, the authors discuss SCP-specific hurdles, including high nucleic acid content, digestibility, and sensory acceptance, as well as the infrastructure and logistics required for industrial-scale production of SCP. Nonetheless, Tazon et al. [169] provide a concrete case study for high-value small molecules by reviewing biotechnological advances in the production of vanillin. Their article demonstrates how PF approaches can outcompete botanical extraction in cost, consistency, and environmental footprint for certain flavor compounds. They detail the metabolic pathways engineered into different microbial hosts, the optimization of precursor supply and cofactor balancing, and the downstream purification steps required to achieve food-grade aroma compounds. This case concretely demonstrates that PF is not limited to macronutrients; it can reliably produce fine and specialty ingredients with a strong sensory impact and economic value. The scientific and technological advances of PF can make it a transformative platform for the next generation of food production. By integrating synthetic biology, metabolic engineering, and advanced bioprocess design, PF enables the tailored biosynthesis of high-value food ingredients, addressing key global challenges in nutrition, sustainability, and resource efficiency.

On the other hand, the impact of PF has also been supported by the development of multiple patents, underscoring how intellectual property protection closely parallels scientific innovation in this field. The patents summarized in Table S1 [155,170,171,172,173,174,175,176,177,178,179,180,181,182,183,184,185,186,187,188,189,190,191,192,193,194,195] show not only the diversity of target compounds but also the trajectory of PF, from early biomass valorization strategies to the production of highly specific recombinant proteins with direct applications in the food industry, as well as the co-production of value-added components. Early innovations, such as the engineering of *Saccharomyces cerevisiae* with amylase and protease activities to valorize food waste for SCP production (US10584359B2), emphasized circular economy and resource recovery. More recent filings, including *Schizochytrium limacinum* engineered for enhanced protein and lipid yield (CN120230649A), highlight the integration of metabolic engineering to improve process efficiency. Beyond bulk biomass, patents on recombinant β-lactoglobulin, ovalbumin, and soy leghemoglobin (WO2020081789A1, WO2016077457A1, US10344304B2/US10273492B2/US10172380B2) illustrate the transition toward high-value proteins with direct applications in food formulations.

In parallel, additional patent families demonstrate the breadth of PF in generating functional ingredients for plant-based foods. Flavor-related patents (US9932610B2/JP6596009B2, WO2021/022216A1, US6372461B1) cover microbial biotransformations for vanillin production, a key aroma compound in the food industry. Natural color patents (US2022/0017878A1, CN119120239A, DK0872554T3) involve engineered microbial systems for carotenoids and retinol derivatives, improving both visual appeal and nutritional quality. Patents on organic acids (US8110381B2, WO2013112939A3, DE10333144B4) showcase microbial synthesis of glutamic, succinic, and citric acids, essential for taste modulation, preservation, and texture. Finally, polysaccharide and texturizer patents (EP3282867B1, US20090093626A1, and WO2021/020995A1, among others) highlight the microbial production of gellan gum, pullulan, hyaluronic acid, and xanthan gum, which are widely used to improve mouthfeel, stability, and processing performance in plant-based formulations. This diversification trend has also been analyzed in broader patent landscape studies. For example, Augustin et al. [27] reported that activity in fermentation between 2000 and 2021 has progressively expanded from basic metabolites toward proteins, lipids, and carbohydrates of direct relevance to the food industry, reflecting a steady shift from commodity compounds to high-value, application-oriented ingredients. Collectively, these patents and scientific research evidence both the historical foundations and the emerging directions of PF in food and ingredient manufacturing, underscoring its potential not only to deliver proteins and essential metabolites but also to provide sensory and functional compounds central to consumer acceptance of alternative foods, particularly by enhancing the nutritional, sensory, and functional performance of plant-based formulations. This is particularly relevant given the limitations of plant proteins, which often require additives and extensive processing to achieve desirable functional and organoleptic properties. In this context, PF offers a sustainable alternative by providing attractive, healthy, safe, and high-value ingredients that reduce reliance on chemical additives and ultra-processed approaches while maintaining quality and supporting the advancement of next-generation plant-based foods.

Despite the advantages of FP, several challenges must be overcome to address current limitations related to process scalability, downstream purification, cost-effectiveness, ingredient functionality, and regulatory approval. Future progress will depend on coupling strain engineering with circular feedstocks, developing scalable fermentation and recovery strategies, and harmonizing regulatory frameworks that support innovation without compromising safety.

## 4. Conclusions and Perspectives

The food industry bears a significant responsibility to meet global food demand with products that are not only nutritious, high-quality, and safe but also sustainable. As highlighted throughout this review, plant matrices and microbial strategies offer a viable and sustainable alternative to developing plant-based foods. The nutritional value of plant proteins and their potential to contribute to food security are analyzed. However, their application in the development of plant-based foods is often limited by the presence of compounds that impart undesirable organoleptic and technological properties. Nevertheless, the application of fermentation technologies, such as TF, BF, and PF constitutes three distinct fermentation approaches that operate through different biological mechanisms and contribute unique functions to the development of alternative protein sources. TF relies primarily on naturally occurring or domesticated microbial communities that act on plant-based substrates to enhance antinutritional factors, digestibility, functional, antimicrobial, sensory and overall consumer acceptance, but it offers limited process control, variable outcomes, and a higher risk of microbial contamination. On the contrary, BF focuses on cultivating microorganisms as the final product, generating high-protein microbial biomass through rapid microbial growth and high cell-density processes; however, it requires controlled bioreactors, higher operational costs, and careful management of nucleic acid content in the final product. Finally, PF differs fundamentally from both, as it employs engineered microorganisms to produce specific target molecules such as pigments, lipids, or other functional compounds that can be incorporated into food formulations to improve their performance and nutritional attributes, but it depends on advanced metabolic engineering and more stringent regulatory frameworks. Although these three approaches have different goals and outputs, they should be viewed as complementary rather than competing technologies with great potential for the development of functional foods and for enhancing the nutritional quality of food made out of alternative protein sources.

The field of alternative protein production continues to grow exponentially, with research efforts clearly reflected in a substantial increase in scientific contributions in recent years. Along these lines, the application of microbial strategies to food production is not the only possible pathway for utilizing plant proteins or single-cell proteins. By-products derived from technological processes, such as supernatants with high biological activity or plant-based wastes rich in compounds undesirable in food applications, may possess properties that can be leveraged by the agricultural, cosmetic, and nutraceutical industries, particularly within the framework of a circular economy. In addition, from an industrial perspective, it is crucial to promote cost efficiency and large-scale production that encompasses life cycle assessments of different production scenarios, allowing the identification of the most influential technical hotspots and challenges for each of the main stages of SCP production. These scientific and industrial dynamics are also reflected in the patent landscape, highlighting growing technological innovation and increasing interest in microbial strategies, side-stream valorization, and PF as sustainable routes for developing novel food ingredients.

On the other hand, recent advancements in the development of advanced genetic and genomic tools and analytical procedures present an opportunity to develop PF for producing specific compounds tailored to food processing challenges, thereby improving the sensory, structural, nutritional, and functional characteristics of new and innovative plant-based foods. In parallel, the rapid expansion of multi-omics approaches is enabling the exploration of novel microorganisms beyond traditional models, positioning them as efficient biofactories that can overcome current limitations. These advances facilitate the selection of microorganisms that are more suitable for the efficient accumulation of target molecules, while ensuring safety for consumption and economic profitability.

The advantages of microbial strategies are increasingly recognized, as reflected in European investment in alternative protein foods research [196,197,198,199,200,201,202,203,204,205,206,207,208,209,210,211,212]. Based on the data shown in Table S2, current trends of the research and innovation projects in EU can be broadly categorized into four main areas: fermentation, circular valorization, mycelium-based proteins, and PF. Among these, fermentation projects predominate both in number and funding, highlighting a strong focus on optimizing microbial processes to enhance flavor, nutritional value, and scalability. Circular bioeconomy approaches, which involve upcycling agricultural and fish by-products into proteins and ingredients, also demonstrate strong support and align with EU sustainability goals. Mycelium-based initiatives are gaining traction, targeting whole-cut meat and seafood analogs as high-value alternatives. Finally, PF, strongly backed by the EIC Accelerator [213], is enabling the production of functional ingredients, such as analogs of egg proteins and designer fats, which can be directly integrated into existing food manufacturing processes. Together, these projects indicate a European shift toward technologically advanced, scalable, and circular solutions for the next generation of plant-based foods. Nevertheless, fermentation technologies face several limitations in the current evidence base. Comparative studies across fermentation modes, substrates, and microbial systems remain limited, and many findings rely on isolated case studies rather than standardized, cross-platform evaluations. Moreover, the scalability, regulatory feasibility, and economic viability of emerging approaches, particularly PF, are still evolving and unevenly documented. For this reason, recognizing these constraints helps contextualize the conclusions of this review and underscores the need for more coordinated research efforts to validate and optimize fermentation strategies for developing next-generation protein ingredients.

In addition to these scientific and technological limitations, broader systemic challenges must also be considered. For instance, despite the clear scientific and economic interest in the alternative protein sector, legislative and regulatory frameworks vary widely across regions. This, combined with the lack of policies promoting its development in low- and middle-income countries, creates uncertainty regarding the true alignment of this sector with sustainability goals. Therefore, it is urgent to adopt measures that ensure that the development of new alternative products is not restricted to specific consumer groups, considering cultural and social differences, enabling their global adoption. Likewise, nutritional needs also extend to the animal sector, particularly pet nutrition, where these novel alternatives may have an even greater likelihood of market entry, given the distinct regulatory requirements compared to human consumption. Based on the previous discussion, continued efforts in these areas will be essential to unlock the full potential of microorganisms in the alternative protein sector and to ensure their successful and sustainable integration into global food systems.

## Figures and Tables

**Table 2:** Microbial species related to desirable odor-forming compounds in traditional fermentation.

Microorganisms	Fermentation System	Metabolic Origin	Chemical Class	Odor Descriptor	References
*Bacillus subtilis* BJ3-2	SSF	Amino acid catabolism and Maillard-type reactions	Pyrazines and other volatile compounds	Roasted, nutty, soy-sauce-like	[102]
*Lacticaseibacillus paracasei*	SmF	Glycolysis and citrate metabolism	Diketones; ketones; lactones	Buttery, creamy, fruity, floral	[103]
*Rhizopus oligosporus* 6010	SSF	Glycolysis, lipid oxidation, amino acid catabolism, esterase activity, and Maillard-like heterocycle formation	Alcohols, aldehydes, phenols, acids, ketones, esters, furans	Citrus/Lemon; Creamy; Fatty; Grass	[104]
*Saccharomyces cerevisiae* RW	SSF	Ester synthesis via acetyl-CoA, higher alcohol formation via Ehrlich pathway, fatty-acid metabolism	Esters; higher alcohols	Fruity, floral, sweet	[105]
*Debaryomyces hansenii* (CGMCC5770)	SSF	Enzymatic release of terpenes and esters; carotenoid degradation; and amino-acid-derived aroma formation	Ester	Minty, wintergreen	[106]
*Candida glabrata* D18	SmF	Release of terpenes via β-glucosidase activity and enhanced ester formation during fermentation	Terpenes, esters, higher alcohols	Floral, fruity (lychee, rose), tropical fruit, sweet	[107]
*Wickerhamomyces anomalus* Y3	SmF or mixed fermentation	Ester biosynthesis, higher alcohol production, alkenes and sulfur compounds via amino acid catabolism	Esters, alcohols, alkenes, disulfides, isothiocyanates	Fruity, floral–honey, wine-like, green, sulfurous	[108]

**Table 3:** Overview of commercial products derived from biomass fermentation strategies to produce alternative protein sources.

Commercial Brand	Microorganisms	Substrates	Fermentation System	Product	Started at	Reference
Quorn	*Fusarium venenatum*	Glucose, NH_4_H_2_PO_4_, Micronutrients	SmF	Mycoprotein-based meat analogs	1985	[143,144]
ENOUGH/Abunda	*Fusarium venenatum*	Glucose, Na_3_C_6_H_5_O_7_, KH_2_PO_4_, NH_4_NO_3_, Micronutrients	SmF	Mycoprotein ingredient	2015	[145]
Nature’s Fynd	*Fusarium flavolapis*	Glucose, NH_4_NO_3_, KH_2_PO_4_, Urea, Micronutrients	SmF	Mycoprotein ingredient	2020	[141]
Solein (Solar Foods)	No published	CO_2_, H_2_, O_2_, Ammonia, minerals (sulfur, phosphorus, calcium, and magnesium)	SmF	Protein-rich powder	2017	[146]
Air Protein	*Xanthobacter autotrophicus*, *Cupriavidus necator*	CO_2_ + H_2_ + Minerals + Air	SmF in gas-fed bioreactor	Protein flour	2019	[147]
The Protein Brewery/Fermotein	*Rhizomucor pusillus*	Glucose syrup (maize), dextrose (wheat), Ammonium salts, Minerals	SmF	Protein ingredient	2020	[148]
MycoTechnology/FermentIQ	*Mushroom mycelia*	Pea, rice protein blends	SSF	Protein powder, functional ingredients	2013	[149,150]
Spirulina (We Are the New Farmers)	*Arthrospira platensis (Spirulina)*	CO_2_, light, mineral medium	Photoautotrophic cultivation	Fresh spirulina paste	2018	[151]
Algama/The Good Spoon	*Chlorella vulgaris*	Sugars, minerals	Heterotrophic fermentation	Eggs substitute	2013	[152]
Sophie’s Bionutrients	*Chlorella vulgaris*	Food waste, spent grains, sugars (molasses), okara	Heterotrophic fermentation	Microalgae protein flour	2019	[153]
Sylpro	*Cyberlindnera jadinii (Torula yeast)*	Agricultural feedstocks	SmF	Single-cell protein (animal feed)	2016	[154]
Yusto				Single-cell protein (food industry)		
Superbrewed Protein	*Clostridium tyrobutyricum*	Agricultural feedstocks	SmF (anaerobic)	Protein-rich ingredient (85%)	2019	[155]

## Data Availability

No new data were created or analyzed in this study.

## Supplementary Materials

The following supporting information can be downloaded at https://www.mdpi.com/article/10.3390/foods15010117/s1, Table S1: Patents on precision fermentation: microorganisms, substrates, culture conditions, and technological highlights. Table S2: European research projects on fermentation strategies for developing alternative protein sources. References [155,170,171,172,173,174,175,176,177,178,179,180,181,182,183,184,185,186,187,188,189,190,191,192,193,194,195,196,197,198,199,200,201,202,203,204,205,206,207,208,209,210,211,212] are cited in the Supplementary Materials.
