# Supplementary material for: Fermentation Technologies to Produce and Improve Alternative Protein Sources
Source: Foods. 2025 Dec 31;15(1):117. doi: 10.3390/foods15010117 (PMC12785468; doi:10.3390/foods15010117)
Supplement: Supplementary file 1 [file foods-15-00117-s001.zip › foods-4018323-supplementary.pdf]

foods

MDPI

Table S1. Patents on precision fermentation: microorganisms, substrates, culture conditions, and technological highlights.

Category	Patent No.	Organism	Substrate/Feedstock	Culture Conditions	Method/Highlights	References
**Protein/ Biomass**	CN120230649A	*Schizochytrium* spLOX-2	Food waste → VFA → SCP	Submerged fermentation; VFA + yeast extract/mineral salts; pH 6–9, 28 °C, 180 rpm	Ferment FW to VFA, then culture *Schizochytrium* for SCP; recombinant strain expresses lactate oxidase to enhance VFA utilization.	[170]
	AU2025201898A1	Gram-positive bacteria (dead, intact cells; ≥60% protein, ≤5% nucleic acids)	Plant fiber substrates (fermentable carbohydrates)	Batch fermentation; plant fiber medium; heat-killed biomass at ≥135 °C	High-protein SCP ingredient (BCAA, Vit B12, minerals); suitable for dairy/meat analogs.	[155]
	US10584359B2	Recombinant *Saccharomyces cerevisiae*	Kitchen waste (starch/protein residues)	Submerged fermentation; engineered yeast with amylase/protease activity	Recombinant yeast engineered with hydrolytic enzymes to directly utilize kitchen waste for protein production.	[171]
	WO2025/065137A1	*Yarrowia lipolytica*	Sugars; agro-industrial by-products	High-density fermentation; optimized nutrient and aeration control	Method for producing single-cell protein via high-density fermentation; improved protein yield and shorter cultivation time.	[172]
	NL2032949B1	*Yarrowia lipolytica, Cutaneotrichosporon oleaginosus*	Biomass-derived substrates	Submerged fed-batch fermentation; mechanical cell disruption	Fermentation of non-pasteurized biomass; oil extraction via mechanical lysis (bead beating, French press, homogenization, screw-press)	[173]
**Flavors**	EP0885968B1	*Streptomyces setonii* (Actinomycetales, Streptomycetaceae)	Ferulic acid (5–40 g/L)	Submerged fermentation; nutrient broth + ferulic acid; pH shift strategy	Biotransformation of ferulic acid → vanillin; guaiacol as by-product; extraction via pH adjustment (>9 guaiacol, ~7 vanillin); solvent methyl-tert-butyl ether; recombinant microbes possible	[174]
	US9932610B2/JP6596009B2	Recombinant *E. coli* expressing VaoA, MtSAD1, AtADH, fcs/ech	Eugenol/ferulic acid	Batch fermentation; recombinant *E. coli* + eugenol feed	Multi-step precision fermentation: VaoA + MtSAD1 convert eugenol → ferulic acid; AtADH enhances aldehyde conversion; ferulic acid → vanillin via fcs and ech pathway.	[175]
	WO2021/022216A1	Modified host cells (*S. cerevisiae, E. coli*)	Glucovanillin, precursors	Batch fermentation; engineered strains; aldehyde dehydrogenase deletions	Strategy to prevent vanillin degradation, increase aldehyde accumulation	[176]

	US6372461B1	Recombinant microbes (*Neurospora, Nocardia*)	Sugars/3-dehydroshikimic acid	Fed-batch fermentation; sugars (3-dehydroshikimic acid)	Route via vanillic acid intermediate; multiple enzymatic steps	[177]
**Naturals colors**	WO2016008779A1	*Monascus* spp. (filamentous fungi)	Sugars (glucose, starch)	Submerged fermentation; hydrocolloid-stabilized medium (starch/gum/gelatin)	Red pigment formulation from *Monascus*, acid-stable for beverages and foods	[178]
	WO2024092372A1	Microbial systems (bacteria/yeast, unspecified)	Plant- or microbe-derived precursors	Fermentation/enzymatic process; optimized downstream purification	Natural-origin food dyes from microbial/plant pathways; focus on regulatory-compliant food applications	[179]
	US8846374B2	*Yarrowia lipolytica* (engineered oleaginous yeast)	Sugars (glucose), lipid precursors	High-cell-density fed-batch; lipid-accumulating medium	Engineered oleaginous yeast expressing carotenoid biosynthesis genes (crtE, crtYB, crtI, etc.) to produce β-carotene, lycopene, lutein, zeaxanthin, canthaxanthin, astaxanthin; co-production strategies (PUFAs + carotenoids).	[180]
	US10227557B2	*Haematococcus pluvialis* (microalga)	CO_2 + light + nutrients (photoautotrophic) or heterotrophic feeds for dark phases	Two-phase photobioreactor cultivation; CO_2 + nutrients; supercritical CO_2 extraction	Process and reactor designs for high-density cultivation and efficient extraction of astaxanthin from Haematococcus; improved productivity via two-phase cultivation and optimized extraction.	[181]
	US6022701A	*Haematococcus pluvialis*	Photoautotrophic media; two-phase cultivation (growth then stress)	Photobioreactor cultivation; nutrient stress induction (cyst stage)	Classic large-scale cultivation protocol for *Haematococcus* with two-phase strategy; downstream concentration/extraction methods.	[182]
	US8288149B2	Oleaginous yeasts/fungi	Sugars/lipid precursors	Fed-batch fermentation; optimized for lipid + carotenoids	Systems for engineered oleaginous yeasts to produce carotenoids; methods for increasing oleaginicity and carotenoid yields; and use of carotenoid-containing biomass in food/feed.	[183]
	US20220017878A1	Engineered microorganisms — unspecified chassis	Sugars; GGPP pathway precursors	Fermentation; pathway engineering (crtE, crtYB, crtl, BCMO, ybbO)	Engineered pathway to produce retinol/derived compounds by enhancing GGPP and carotenoid cleavage steps (shows route from carotenoids to retinol). Relevant to carotenoid → vitamin A/retinol value-added products via precision fermentation.	[184]
	CN119120239A	*Yarrowia lipolytica* (engineered)	Sugars; lipid precursors	Fermentation; *Yarrowia* knockouts/overexpression; lipid precursor medium	Genetic engineering of *Y. lipolytica* to overproduce retinol/carotenoids; fermentation engineering details included (industrial fermentation focus).	[185]

	DK0872554T3	Recombinant bacteria expressing *Flavobacterium* carotenoid enzymes	Sugars/fermentation carbon sources	Bacterial fermentation; heterologous carotenoid enzymes	Production of canthaxanthin, adonixanthin, astaxanthin, and zeaxanthin via transformed cells	[186]
**Organic acids**	US 8110381B2	*Corynebacterium glutamicum* (gluX mutant)	Sugars (glucose)	Fed-batch fermentation	Inactivation of gluX gene to increase L-glutamate accumulation	[187]
	CN104178438B	*Lactobacillus delbrueckii*	Molasses (sugar feed)	Fermentation with controlled pH; optimized nutrients	Industrial-scale method for high-purity L-lactic acid; adapted to molasses feedstock	[188]
	EP1096020B1	*Aspergillus niger*	Sucrose/glucose (starch hydrolysates)	Bubbled-column or airlift submerged fermentation; mineral-anion-free nitrogen source; pH and aeration controlled	Citric acid manufacture with downstream: cell removal, UF, activated carbon, cation exchange, crystallization	[189]
**Polysaccharides and texturizers**	EP3282867B1	*Sphingomonas elodea* (wild-type and mutant strains)	Sugars (glucose, sucrose), nitrogen, minerals	Aerobic submerged fermentation; controlled pH (6–7) and temperature (~28–30 °C) in bioreactor	Industrial process for gellan gum production; includes downstream purification, control of gel strength and granule density	[190]
	US20090093626A1	*Xanthomonas campestris* (recombinant; overexpression of gumB/gumC)	Carbohydrates (e.g., sucrose/glucose)	Aerobic submerged fermentation; aerated and stirred bioreactor; downstream alcohol precipitation	Genetic strategy to increase intrinsic and seawater viscosity; viscosity control discussed (pasteurization)	[191]
	US5019514A	*Aureobasidium pullulans* (mutant strain)	Glucose/sucrose (carbohydrate feed)	Aerobic submerged fermentation; pH control; nutrient salts	Strain engineering for reduced melanin; standard purification (filtration, precipitation)	[192]
	AU2018253392A1	*Aureobasidium pullulans*	Liquefied starch/glucose–sucrose carbohydrate media	Aerobic submerged fermentation with aeration/agitation (sparging); 25–30 °C, ~3–7 days; standard downstream recovery	Process controls for consistent molecular weight and low mono/di/oligosaccharides; guidance on media and aeration for robust pullulan formation	[193]
	WO2021020995A1	Engineered *Bacillus subtilis* (hasA fusion + host genes)	Glucose/carbohydrate feed	Submerged fermentation: pH/oxygen controlled	Genetically engineered *Bacillus* produces HA (non-pathogenic host); suitable for scalable, food/cosmetic-grade HA	[194]
	WO2012030651A1	*Xanthomonas campestris*	Sugars/biomass-derived carbohydrates	Aerobic submerged fermentation	Fast hydration/high viscosity xanthan; properties enhanced vs. conventional gum; maintains shear/enzyme stability	[195]

Table S2. European research projects on fermentation strategies for developing alternative protein sources.

Project name	Description	Programs	Coordinated by	Funding Scheme	EU contribution	Period	Reference
Production of superior meat analogs by bridging the benefits of plant proteins and mycelium protein biomass produced from circular substrates (PLANTO-MYC)	Explores fungal mycelium as a new sustainable protein source; aims to upscale mycoprotein ingredients for mainstream food applications.	HORIZON.2.6 - Food, Bioeconomy, Natural Resources, Agriculture, and Environment.	RISE PROCESSUM AB (Sweden)	HORIZON Research and Innovation Actions (RIA)	4.618.367,50 €	01/01/2025 - 31/12/2028	[196]
Impact of alternative protein sources to improve nutrition (PROTEIN4IMPACT)	Designs functional protein ingredients from unconventional sources, agrifood, and fisheries by-products for healthier, sustainable food systems; targets broad industrial applications.	HORIZON.2.6 - Food, Bioeconomy, Natural Resources, Agriculture, and Environment	CENTRALE-SUPELEC (France)	HORIZON-RIA	5.273.375,00 €	01/01/2025 - 31/12/2027	[197]
Propelling health and sustainability through innovative food products and processes (Sustain-a-bite)	Applies efficient bioprocessing to valorize side-streams and waste to produce new plant-based food ingredients, enhancing the circular bioeconomy and economic competitiveness of plant-based ingredients.	HORIZON.2.6 - Food, Bioeconomy, Natural Resources, Agriculture, and Environment	Technical Research Centre of Finland VTT (Finland)	HORIZON-RIA	4.996.972,25 €	01/10/2024 - 31/03/2028	[198]
Unleashing the flavor potential of plant-based foods via fermentation (FlavourFerm)	Optimizes precision fermentation, biomass fermentation, and traditional fermentation to enhance the flavor and nutrition of plant-based foods.	HORIZON.2.6 - Food, Bioeconomy, Natural Resources, Agriculture, and Environment	University of Southern Denmark (Denmark)	HORIZON Innovation Actions (IA)	4.484.546,98 €	01/11/2024 - 30/04/2028	[199]
Green VALORisation cascade approach of fISH waste and by-products through fermentation towards a zero-waste future (VALORISH)	Applies a cascade biorefinery approach to fish waste, creating protein-rich ingredients and bio-products for sustainable food production.	HORIZON.2.6 - Food, Bioeconomy, Natural Resources, Agriculture, and Environment.	Idener Research and Development (Spain)	HORIZON-RIA	3.935.845,00 €	01/05/2024 - 31/10/2027	[200]

Flavor, odour and texture improvements of plant-based dairy products using microbial fermentation products (DELICIOUS)	Develops biotechnological solutions to improve taste and nutritional value of plant-based foods; combines fermentation with sensory research.	HORIZON.2.6 - Food, Bioeconomy, Natural Resources, Agriculture, and Environment	RISE PROCESSUM AB (Sweden)	HORIZON-IA	4.499.800,88 €	01/11/2024 - 31/10/2028	[201]
Alternative sources for high added value food and/or feed ingredients (SYLPLANT)	Focuses on building a sustainable plant to valorize underused feedstock to produce protein-rich ingredients by yeast fermentation for the food and feed markets.	HORIZON.2.6 - Food, Bioeconomy, Natural Resources, Agriculture, and Environment. HORIZON.2.6.6 - Bio-based Innovation Systems in the EU Bioeconomy	ARBIOM (France)	HORIZON JU Innovation Actions (JU-IA)	13.909.015,08 €	01/06/2023 - 31/05/2026	[202]
New sustainable proteins for food, feed and non-food bio-based applications (InnoProtein)	Works on biorefinery and microbial fermentation to create diverse alternative protein sources with lower environmental impact.	HORIZON.2.6 - Food, Bioeconomy, Natural Resources, Agriculture, and Environment. HORIZON.2.6.6 - Bio-based Innovation Systems in the EU Bioeconomy	FUNDACION TECNALIA (Spain)	HORIZON JU Research and Innovation Actions (JU-RIA)	4.592.391,64 €	01/06/2023 - 31/05/2027	[203]
Innovative, sustainable, and circular production of purple phototrophic bacteria as health-promoting ingredient for food and feed applications (Purple4Life)	Harnesses plant pigments and antioxidants to develop functional food ingredients with added health value.	HORIZON.2.6 - Food, Bioeconomy, Natural Resources, Agriculture, and Environment. HORIZON.2.6.6 - Bio-based Innovation Systems in the EU Bioeconomy	UNIVERSITE DE MONS (Belgium)	HORIZON-JU-RIA	3.721.693,75 €	01/06/2025 - 31/05/2029	[204]
Alternative PROteins from MIcrobial fermentation of non-conventional SEA sources for Next-Generation food, feed and non-food bio-based applications (PROMISEANG)	Utilizes biomass fermentation, solid-state, and submerged processes to convert marine waste into novel protein ingredients, addressing both functionality and sustainability.	HORIZON.2.6 - Food, Bioeconomy, Natural Resources, Agriculture, and Environment. HORIZON.2.6.6 - Bio-based Innovation Systems in the EU Bioeconomy	Universidad de Vigo (Spain)	HORIZON-JU-RIA	4.535.097,50 €	01/09/2023 - 31/08/2027	[205]
Valorization of Agro-Industrial Waste through Fungi Fermentation supported by Digital Modeling (ZEST)	Develops fungal protein from agricultural residues using advanced modeling; demonstrates circularity and scalability.	HORIZON.2.6 - Food, Bioeconomy, Natural Resources, Agriculture, and Environment. HORIZON.2.6.6 - Bio-based Innovation Systems in the EU Bioeconomy	Danish Technological Institute (Denmark)	HORIZON-JU-IA	5.931.246,25 €	01/06/2024 - 31/05/2028	[206]

Innovative pulse and cereal-based food fermentations for human health and sustainable diets (HealthFerm)	Investigates health-focused fermentations to develop nutritious plant-based foods with probiotic properties.	HORIZON.2.6 - Food, Bioeconomy, Natural Resources, Agriculture and Environment. HORIZON.2.6.5 - Food Systems	KU Leuven (Belgium)	HORIZON-RIA	11.303.944,75 €	01/09/2022 - 31/08/2026	[207]
Transforming Agri-Food By-Products into High-Nutritional Value, Sustainable Proteins and Ingredients (MOA FOODTECH)	Uses AI-driven fermentation to upcycle agro-food side-streams into customised proteins and ingredients.	HORIZON.3.1 - The European Innovation Council (EIC)	MOA Biotech Sl (Spain)	HORIZON EIC Accelerator	2.258.016,00 €	01/06/2025 - 31/05/2027	[208]
Pioneering vegan whole cuts through mycelium solid-state fermentation (Esencia Foods)	Develops whole-cut meat and seafood analogs from mycelium using solid-state fermentation.	HORIZON.3.1 - The European Innovation Council (EIC)	Esencia Foods Spain S.L. (Spain)	HORIZON EIC Accelerator	2.020.666,81 €	01/06/2025 - 31/05/2027	[209]
Inspiring CO2 circularity by introducing carbon transformation to our plates (C-2C - Protein)	Develops functional food ingredients for human consumption through hybrid vertical microalgae fermentation technology using CO2 as a carbon source.	HORIZON.3.1 - The European Innovation Council (EIC)	Solmegia Monoprosopi I.K.E (Greece)	HORIZON EIC Accelerator	2.442.727,56 €	01/09/2024 - 31/08/2026	[210]
Novel precision fermentation process to produce animal-free bioidentical ovalbumin (Bioalbumen)	Produces bioidentical ovalbumin via precision fermentation, a sustainable replacement for egg white protein.	HORIZON.3.1 - The European Innovation Council (EIC)	ONEGO BIO LTD (Finland)	HORIZON EIC Accelerator	2.499.999,00 €	01/01/2025 - 31/12/2026	[211]
Harnessing the immense potential of precision fermentation to produce animal fats for the next generation of meat and dairy alternatives (Melt and Marble)	Designs precision-fermented fats to improve texture, flavor, and functionality in plant-based foods.	HORIZON.3.1 - The European Innovation Council (EIC)	Melt&Marble (Sweden)	HORIZON EIC Accelerator	2.485.840,00 €	01/01/2025 - 30/06/2026	[212]
